# Atmospheric H_2_S: Impact on Plant Functioning

**DOI:** 10.3389/fpls.2019.00743

**Published:** 2019-06-11

**Authors:** Ties Ausma, Luit J. De Kok

**Affiliations:** Laboratory of Plant Physiology, Groningen Institute for Evolutionary Life Sciences, University of Groningen, Groningen, Netherlands

**Keywords:** air pollution, hydrogen sulfide, sulfur metabolism, glutathione, *Brassica*

## Abstract

Hydrogen sulfide (H_2_S) is an air pollutant present at high levels in various regions. Plants actively take up H_2_S via the foliage, though the impact of the gas on the physiological functioning of plants is paradoxical. Whereas elevated H_2_S levels may be phytotoxic, H_2_S levels realistic for polluted areas can also significantly contribute to the sulfur requirement of the vegetation. Plants can even grow with H_2_S as sole sulfur source. There is no relation between the rate of H_2_S metabolism and the H_2_S susceptibility of a plant, which suggests that the metabolism of H_2_S does not contribute to the detoxification of absorbed sulfide. By contrast, there may be a strong relation between the rate of H_2_S metabolism and the rate of sulfate metabolism: foliar H_2_S absorbance may downregulate the metabolism of sulfate, taken up by the root. Studies with plants from the *Brassica* genus clarified the background of this downregulation. Simultaneously, these studies illustrated that H_2_S fumigation may be a useful tool for obtaining insight in the regulation of sulfur homeostasis and the (signal) functions of sulfur-containing compounds in plants.

## Introduction

Hydrogen sulfide (H_2_S) is a gaseous compound present in the global atmosphere (Watts, 2000). Together with sulfur dioxide (SO_2_) and a variety of organo-sulfur gases, H_2_S plays a pivotal role in shaping the earth’s climate (Sipilä et al., 2010; Perraud et al., 2015). The gas naturally originates from volcanoes and geothermal vents as well as from wetlands, salt marshes and estuaries, where it is produced by bacteria during the anaerobic decay of organic sulfur compounds (Kanda and Tsuruta, 1995; Watts, 2000; Stern, 2005). The estimated natural emission of H_2_S is 4.4 Tg per year, which is only a small fraction of the total natural sulfur gas emission, estimated at 52 Tg per year (Watts, 2000). SO_2_ and dimethylsulfide (DMS) make up the majority of this emission: 23 and 24.5 Tg per year, respectively (Watts, 2000; Carn et al., 2017). Besides from natural sources, atmospheric H_2_S originates from livestock production and industrial processes, such as the combustion of biomass and fossil fuels (Watts, 2000). The anthropogenic emission of sulfur gases is exceeding the natural sulfur gas emission and is currently estimated at 70–100 Tg per year (Klimont et al., 2013; Fioletov et al., 2016). Most of this sulfur is emitted as SO_2_, though approximately 3 Tg per year is emitted as H_2_S (Klimont et al., 2013; Fioletov et al., 2016).

The residence time of emitted H_2_S in the atmosphere is short (approximately 15 days), since H_2_S is rapidly oxidized by hydroxyl radicals and other atmospheric oxidants to SO_2_ and finally sulfate (Trudinger, 1986). Consequently, in rural areas, H_2_S concentrations are ranging between 0.02 and 0.3 nl L^−1^ (Kellogg et al., 1972; Slatt et al., 1978; Beauchamp et al., 1984). However, in regions with volcanic activity and in regions with polluting industrial or livestock production, H_2_S concentrations may easily surpass the odor threshold level of 0.02 μl L^−1^ (resulting in a distinct rotten egg smell; Beauchamp et al., 1984). Moreover, in the close vicinity of volcanoes and geothermal wells, atmospheric H_2_S concentrations may even exceed 0.1 μl L^−1^ (Ernst, 1997; Schulte et al., 1997; Baillie et al., 2016).

The impact of elevated H_2_S levels on plants is paradoxical. Although high atmospheric H_2_S concentrations may negatively affect plant growth and survival, the foliar uptake of H_2_S may also substantially contribute to plant sulfur nutrition (De Kok, 1990; De Kok et al., 2007). This concise review presents an overview of the impact of atmospheric H_2_S on the physiological functioning of plants.

## Uptake and Emission of H_2_S by Plants

Atmospheric H_2_S may be adsorbed at the leaf’s surface, though H_2_S uptake mainly occurs via stomata: the cuticle is hardly permeable for gases (Lendzian, 1984). The rate of foliar gas uptake can be described by Fick’s law of diffusion: *J* = Δ*c* × *g*. In this equation, *J* represents the rate of gas uptake by the shoot (μmol cm^−2^ s^−1^), Δ*c* the concentration gradient of the gas between the atmosphere and the shoot’s interior (μmol cm^−3^), and *g* the diffusive conductance of the shoot to the gas (cm s^−1^; Nobel, 1983; De Kok and Tausz, 2001; De Kok et al., 2007). The diffusive conductance of the shoot is predominantly determined by stomatal conductance and mesophyll conductance (Nobel, 1983; De Kok and Tausz, 2001; De Kok et al., 2007). Whereas stomatal conductance depends on the extent of stomatal aperture, mesophyll conductance depends on the physical and biochemical characteristics of a gas, *viz.* its solubility in the aqueous phase of the mesophyll, its reactivity with cellular components and in case of some gases (e.g., CO_2_) its rate of metabolism (Nobel, 1983; De Kok and Tausz, 2001; De Kok et al., 2007). The diffusive conductance of the shoot is frequently expressed in mmol m^−2^ s^−1^. For conversion to cm s^−1^, the volume of 1 mol of a gas at 1 atm and 20°C is 24.06 dm^3^ (Nobel, 1983). This corresponds to 41.6 mol m^−3^. Therefore, a gas conductance of 1 cm s^−1^ at 1 atm and 20°C equals 41.6 mol m^−3^ × 0.01 m s^−1^ = 416 mmol m^−2^ s^−1^.

The uptake of H_2_S by plant shoots follows distinct kinetics, which greatly differ from the kinetics observed for other sulfur gases. For instance, SO_2_ uptake rates generally increase linearly with atmospheric SO_2_ concentration (Tausz et al., 1998; Van der Kooij and De Kok, 1998; De Kok and Tausz, 2001). Stomatal conductance is limiting SO_2_ uptake rates, since the diffusive conductance of the shoot to SO_2_ is often close to the stomatal conductance for water vapor (Tausz et al., 1998; Van der Kooij and De Kok, 1998; De Kok and Tausz, 2001). In accordance with this, the mesophyll conductance to SO_2_ is high and therefore not limiting uptake rates. SO_2_ has a high solubility in the aqueous phase of the mesophyll: it has a rather high Henry’s law solubility constant of 1.23 M/atm at 25°C (De Bruyn et al., 1995). Moreover, it rapidly reacts with mesophyll water, resulting in the formation of sulfurous acid (Tausz et al., 1998).

**Figure 1:**
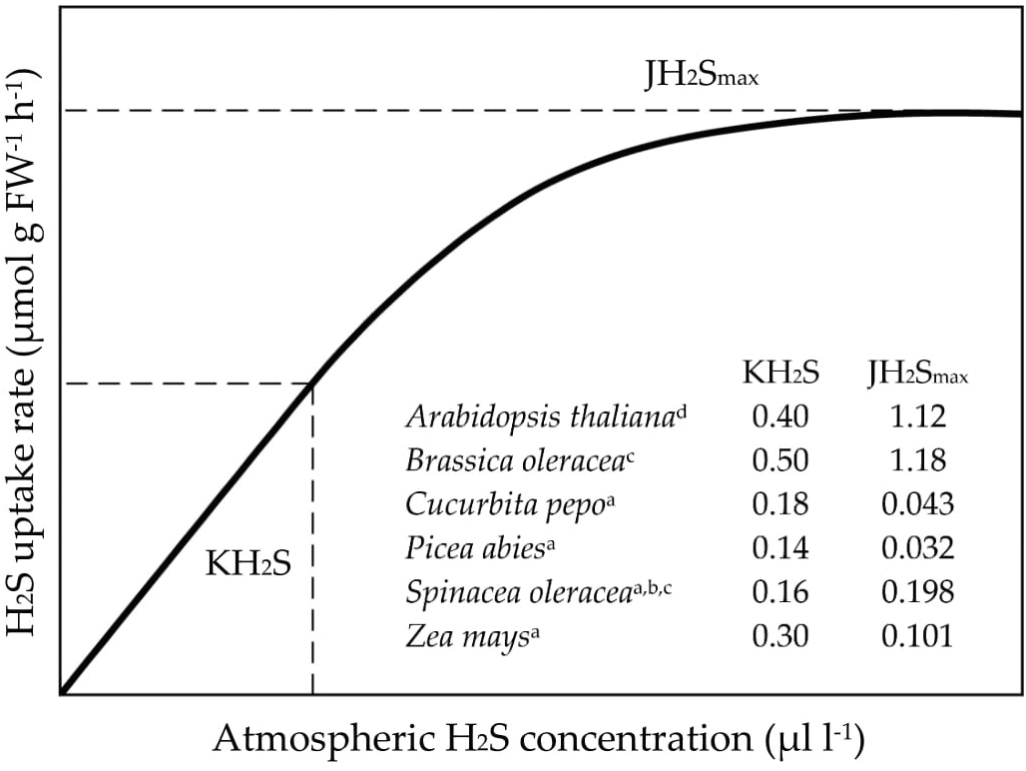
The kinetics of foliar H_2_S uptake. JH_2_S_max_ represents the maximum uptake rate of H_2_S and KH_2_S the concentration at which ½JH_2_S_max_ is reached. FW: fresh weight. Figure modified after De Kok et al. (2002). Data derived from De Kok et al. (1989^a^, 1991^b^, 1997^c^) and Van der Kooij and De Kok (1998^d^).

In contrast to SO_2_, H_2_S uptake rates follow saturation kinetics with respect to the atmospheric H_2_S level (Figure 1; De Kok et al., 2002, 2007, 2009). These kinetics, which can be described by the Michaelis-Menten equation, are controlled by mesophyll conductance (Figure 1). At non-saturating atmospheric H_2_S levels, uptake rates increase linearly with external H_2_S concentration, since stomatal conductance is not affected upon exposure to H_2_S concentrations <1 μl L^−1^ (De Kok et al., 1989, 1991, 1997). However, at saturating atmospheric H_2_S levels, mesophyll conductance prevents further increments in H_2_S uptake rates (De Kok et al., 1989, 1991, 1997).

It is evident that at the pH of mesophyll cells (between 5 and 6.4) absorbed H_2_S remains largely undissociated (H_2_S → HS^−^ + H^+^; p*K*_a_ = 7.0), causing it to easily pass cellular membranes (Cope and Spedding, 1982; Mathai et al., 2009; Riahi and Rowley, 2014). H_2_S is only slightly soluble in the mesophyll: it has a Henry’s law solubility constant of 0.086 M/atm at 25°C (De Bruyn et al., 1995). However, mesophyll conductance appears to be strictly controlled by the rate of sulfide metabolism in the amino acid cysteine (De Kok et al., 1989, 1991, 1998). After absorbance, H_2_S is incorporated with very high affinity in cysteine via the reaction of sulfide with *O*-acetylserine (OAS), catalyzed by the enzyme *O*-acetylserine(thiol)lyase (OAS-TL; De Kok et al., 2007, 2009). The activity of OAS-TL, the affinity of the enzyme for sulfide and the availability of OAS determine mesophyll conductance (De Kok et al., 2007, 2009). Consequently, in spinach (*Spinacia oleracea*) H_2_S uptake rates were, in contrast to SO_2_ uptake rates, strongly dependent on shoot temperature with lower uptake rates at lower temperatures (De Kok et al., 1991). Moreover, in spinach, the maximum H_2_S uptake rate (JH_2_S_max_) could be enhanced by the direct supply of OAS to foliar tissue (Buwalda et al., 1992). Notably, a second cysteine-producing reaction may further determine mesophyll conductance. Cysteine desulfhydrases (DES) have significance in the degradation of cysteine, which results in the endogenous release of sulfide (Schütz et al., 1991). However, circumstantial evidence suggests that the reverse reaction may be relevant for foliar H_2_S absorbance: in leaf homogenates of cucurbit plants (*Cucurbita pepo* spp.) DES assimilated atmospheric H_2_S in cysteine by using ammonia and pyruvate as substrates (Schütz et al., 1991). Nevertheless, the significance of this reaction for intact plants remains to be studied (De Kok et al., 1998, 2007).

Maximum H_2_S uptake rates and KH_2_S values (*viz.* the H_2_S concentration at which ½JH_2_S_max_ is reached) differ considerably among species (Figure 1; De Kok et al., 2002). For example, whereas measured KH_2_S values ranged from 0.14 to 0.50 μl L^−1^ H_2_S, maximum H_2_S uptake rates varied between 0.03 and 1.18 μmol H_2_S g FW^−1^ h^−1^ (Figure 1). This variation corresponded with variation in the rate of sulfide incorporation in cysteine (De Kok et al., 2007). However, it remains elusive to what extent it also coincides with variation in sulfur growth requirement among species. It is unknown if, for instance, species with a high sulfur demand also have high H_2_S uptake efficiencies.

Besides H_2_S uptake, plants may also emit H_2_S. It has been suggested that emission of H_2_S to the atmosphere has significance in regulating sulfur homeostasis (Schröder, 1993). Plants may, temporarily, emit elevated levels of H_2_S via their foliage into the atmosphere when exposed to excess sulfur in the form of SO_2_, sulfate or cysteine (Rennenberg, 1984; De Kok, 1990; Schröder, 1993; Bloem et al., 2007). For instance, depending on the atmospheric SO_2_ level, up to 15% of foliarly absorbed SO_2_ could be re-emitted as H_2_S (De Kok, 1990). However, to what extent H_2_S evolution has significance in regulating sulfur homeostasis in the absence of excess sulfur is unclear. If plants were grown with a normal sulfur supply, the H_2_S evolution rate generally constituted a negligible fraction of the total sulfur assimilation rate (Stulen and De Kok, 1993). For instance, in spruce (*Picea abies*), this fraction was less than 0.1% (Rennenberg et al., 1990). More likely, the rate of foliar H_2_S emission just reflects the rate of sulfate reduction as well as the activity and sulfide-affinity of the cysteine synthesizing and degrading enzymes (*viz.* OAS-TL and DES, possibly together with other enzymes; Hell et al., 2002). Irrespective of this, minute H_2_S emission levels might still have physiological significance in, for instance, plant stress protection. Emitted H_2_S might possibly degrade leaf surface ozone (O_3_; Schnug, 1997; Haneklaus et al., 2007). Furthermore, H_2_S emission may have importance in the defense of plants against pathogen attacks (Bloem et al., 2007; Haneklaus et al., 2007).

## Phytotoxicity of H_2_S

The foliar absorbance of atmospheric H_2_S may negatively affect plant functioning. H_2_S is a very reactive compound and, similar to cyanide, it complexes with high affinity to the metallo-groups in proteins (Mudd, 1979; Beauchamp et al., 1984; Martin and Maricle, 2015). In several plants, exposure to H_2_S inhibited respiration, which could be explained by the reaction of H_2_S with the heme-group of cytochrome *c* oxidase (Martin and Maricle, 2015). Additionally, in various plants the presence of H_2_S repressed the activity of a broad group of (likely heme-containing) NADH-oxidizing enzymes (Maas and De Kok, 1988). Notably, these repressions may directly result in a lower plant growth and survival, but also indirectly: by disturbing energy homeostasis, H_2_S presence may increase the susceptibility of plants for other environmental stressors. For instance, exposure to 0.25 μl L^−1^ H_2_S decreased the freezing tolerance of the foliage of winter wheat (*Triticum aestivum*; Stuiver et al., 1992).

H_2_S presence may also affect the activity of enzymes involved in photosynthetic CO_2_ fixation and photosynthetic electron transport (Oliva and Steubing, 1976; Coyne and Bingham, 1978; De Kok et al., 1983a; Taylor and Selvidge, 1984). In isolated spinach chloroplasts H_2_S exposure inhibited the photoreduction of NADP^+^ and upon illumination it initiated oxygen uptake by the chloroplasts (De Kok et al., 1983a). Sulfide-induced oxygen uptake by chloroplasts was sensitive to the herbicide DCMU (3-(3,4-dichlorophenyl)-1,1-dimethylurea) and prevented by the addition of superoxide dismutase to the chloroplast suspension. This indicated that sulfide was oxidized by chloroplasts, its oxidation being initiated by superoxide formed upon illumination at the reducing side of photosystem I (De Kok et al., 1983a). Nevertheless, reductions in photosynthesis are unlikely to be the primary basis of H_2_S toxicity, since generally in intact plants photosynthesis was only reduced after prolonged exposure to toxic H_2_S concentrations (Maas et al., 1987a, 1988; De Kok, 1989, 1990).

There is a large variation in H_2_S susceptibility between species as well as between cultivars of the same species. Whereas continuous exposure to 0.03 μl L^−1^ H_2_S (a level realistic for regions with industrial or agricultural pollution) negatively affected the growth of susceptible plants (e.g., various spinach cultivars), it stimulated the growth of several other plants on sulfur-sufficient soils [e.g., lettuce (*Lactuca sativa*), alfalfa (*Medicago sativa*), and sugar beet (*Beta vulgaris*); Thompson and Kats, 1978; De Kok, 1989, 1990]. Moreover, whereas in some plants (e.g., lettuce, sugar beet, and common grape vine; *Vitis vinifera*), visible leaf injury developed upon prolonged exposure to 0.3 μl L^−1^ H_2_S (a level realistic for areas nearby, e.g., volcanoes), other plants remained unaffected (Thompson and Kats, 1978; De Kok, 1989, 1990). A small number of plants could actually tolerate H_2_S levels as high as 20 μl L^−1^ [e.g., maiden silvergrass (*Miscanthus sinensis*), which inhabits zones very close to volcanoes; Mudd, 1979]. However, at higher H_2_S levels, all plants quickly developed severe leaf necrosis and rapidly started wilting (Mudd, 1979).

Variation in susceptibility to the negative effects of H_2_S may partly be associated with differences in the impact of H_2_S on energy homeostasis. The *in vitro* cytochrome *c* oxidase activity was less affected by H_2_S exposure in flooding-tolerant than in flooding-sensitive species (Martin and Maricle, 2015). Flooding can induce H_2_S formation in soils, which may explain this variation (Martin and Maricle, 2015). Furthermore, in spinach, the more susceptible a plant was for H_2_S, the more the *in vitro* NADH oxidation capacity of shoots was decreased upon H_2_S exposure (Maas and De Kok, 1988). In addition, in experiments with other plant species in which growth was not affected by H_2_S presence, also the *in vitro* NADH-oxidation capacity was not affected (Stulen et al., 1990). Differences in H_2_S tolerance could also be related to differences in plant morphology. In general, dicots appeared to be more susceptible to atmospheric H_2_S than monocots (Stulen et al., 1990, 2000). In monocots, H_2_S can hardly penetrate the shoot meristem, because the meristem is sheltered by a whorl of leaves (Stulen et al., 1990, 2000). This may cause monocots to be relatively H_2_S tolerant, since carefully uncovering the shoot meristem of maize (*Zea mays*) increased its susceptibility to H_2_S (Stulen et al., 1990, 2000). Whereas this uncovering did not affect the elongation rate of leaves, it delayed the initial leaf development from the meristem and it triggered cell deformations as well as chromosomal irregularities inside the meristem (Stulen et al., 1990, 2000). Notably, it remains elusive if other morphological traits (e.g., traits associated with leaf anatomy) also explain variation in H_2_S phytotoxicity. Furthermore, it remains elusive if life-history traits (e.g., being annual or perennial) contribute to this variation. However, by contrast, it has been observed that variation in H_2_S phytotoxicity is not directly interrelated to the capacity of a plant to metabolize sulfide in organic compounds (see the next section).

## Impact of H_2_S on Plant Sulfur Metabolism

Besides reducing growth and survival, plants may also benefit from the presence of atmospheric H_2_S. Since sulfide is a substrate for cysteine synthesis, the gas can be used to synthesize proteins and other organic compounds (De Kok et al., 2007, 2009; Koralewska et al., 2008). Plants can even grow with atmospheric H_2_S as the only sulfur source (*viz.* in the absence of root sulfate supply; Buchner et al., 2004; Aghajanzadeh et al., 2016). Atmospheric H_2_S levels of 0.06 μl L^−1^ were already sufficient to fully cover the organic sulfur requirement of curly kale (*Brassica oleracea*; Buchner et al., 2004). Since curly kale is characterized by an extraordinary high sulfur demand, H_2_S levels realistic for polluted regions (e.g., regions with volcanic activity or intensive animal farming) may significantly contribute to the sulfur requirement of plants in general (Aghajanzadeh et al., 2014, 2015, 2016).

Typically, a significant part of absorbed H_2_S (up to 30%) can be revealed in plants as water-soluble non-protein thiols (De Kok, 1990; Poortinga and De Kok, 1997). In shoots the content of these metabolites rapidly and strongly increases upon H_2_S exposure (De Kok et al., 1983b, 1985; Maas et al., 1985; Poortinga and De Kok, 1997; Riemenschneider et al., 2005; Shahbaz et al., 2013). In the absence of atmospheric H_2_S, water-soluble non-protein thiols constitute 2–4% of total sulfur present in tissues (Noctor et al., 2012). Generally, glutathione is the most abundant water-soluble non-protein thiol with its content accounting for more than 90% of the total water-soluble non-protein thiol pool (Noctor et al., 2012). Glutathione is synthesized from cysteine in a two-step process. Cysteine first reacts with glutamate to yield γ-glutamylcysteine, which subsequently reacts with glycine to yield glutathione (Hawkesford and De Kok, 2006).

Depending on the species and cultivar, H_2_S exposure increased shoot water-soluble non-protein thiol levels up to five-fold (De Kok et al., 1983b, 1985; Maas et al., 1985; Poortinga and De Kok, 1997; Riemenschneider et al., 2005; Shahbaz et al., 2013). Generally, thiol accumulations were stronger at higher atmospheric H_2_S levels and the accumulations were not affected by plant age, exposure temperature or the applied light regime (De Kok, 1990). Thiol levels often started to increase within one or 2 h after the onset of H_2_S exposure and maximum thiol levels were usually reached after one or 2 days of exposure, independent from the applied H_2_S concentration (De Kok et al., 1985; Maas et al., 1987b,c; Stuiver et al., 1992; Poortinga and De Kok, 1997). Generally, thiol accumulations could not solely be attributed to enhanced glutathione levels. For instance, a 12-h exposure of different plant species to 0.25 μl L^−1^ H_2_S resulted not only in a two- to three-fold increase in shoot glutathione content, but also in a 8- to 37-fold increase in shoot cysteine content (Buwalda et al., 1988, 1993, 1994). Additionally, in the dark, H_2_S exposure resulted in an accumulation of γ-glutamylcysteine (up to 20-fold) due to a limitation in glycine availability, which was caused by the absence of photorespiration in the dark (Buwalda et al., 1988, 1993). However, upon transition to the light, accumulated γ-glutamylcysteine rapidly disappeared as it was metabolized into glutathione (Buwalda et al., 1988, 1993).

Besides altering shoot thiol pools, H_2_S exposure occasionally also alters root thiol pools. However, upon H_2_S fumigation root thiol pools usually increased significantly less than shoot thiol pools (maximum two-fold) and the increases could often fully be ascribed to enhanced glutathione levels (De Kok et al., 1997; Poortinga and De Kok, 1997; Stuiver and De Kok, 1997; Tausz et al., 1998; Westerman et al., 2000a). In general, glutathione is predominantly present in plants in its reduced form (e.g., in spinach leaves >84%; De Kok et al., 1986). In spinach, the ratio between reduced and oxidized glutathione as well as the activity of glutathione reductase were not substantially altered by exposure to 0.25 μl L^−1^ H_2_S (De Kok et al., 1986; Tausz et al., 1998).

After termination of H_2_S exposure, thiol levels decrease. In spinach, cysteine and glutathione levels in shoots decreased simultaneously (De Kok et al., 1985, 1986; Maas et al., 1987b; Buwalda et al., 1994). Moreover, a transition from dark to light immediately after the end of H_2_S exposure resulted in a decrease in accumulated γ-glutamylcysteine and a simultaneous increase in glutathione, after which both cysteine and glutathione contents decreased at similar rates (Buwalda et al., 1994). Independent from the H_2_S concentration that plants were exposed to, thiol levels restored to the levels of unexposed plants within 1 or 2 days (Buwalda et al., 1994). In spinach, decreases in thiol levels were not associated with the foliar emission of H_2_S, indicating that desulfhydration of cysteine was not involved in the loss of thiols (De Kok, 1989, 1990). Apparently, accumulated thiols were rapidly metabolized in other compounds.

It is yet unclear why H_2_S exposure strongly enhances the size of the thiol pool. H_2_S may partly be metabolized in cysteine and glutathione in another subcellular compartment than sulfate taken up by the root (*viz.* in the mitochondria and/or cytosol instead of the chloroplasts). In this way, the metabolism of atmospheric H_2_S could be beyond the control of existing regulatory feedback mechanisms (Hesse et al., 1997; Saito et al., 1997).

It may be hypothesized that variation in the rate of sulfide incorporation in cysteine, glutathione, and other organic compounds (partly) explains variation in H_2_S tolerance between plants. However, there is no relation between the H_2_S uptake kinetics and the H_2_S susceptibility of plants (*viz.* H_2_S phytotoxicity; De Kok et al., 1989, 1998, 2002). Moreover, whereas in thale cress (*Arabidopsis thaliana*) OAS-TL knock-out mutants differed in leaf cysteine and glutathione concentrations from wild-type plants (when exposed to atmospheric H_2_S concentrations up to 1 μl L^−1^), leaf sulfide concentrations and sulfide tolerance were not different (Birke et al., 2015). Seemingly, the metabolism of sulfide in organic compounds is not involved in the detoxification of absorbed H_2_S. It is thus unlikely that differences in the capacity to metabolize H_2_S explain differences in the phytotoxicity of H_2_S.

It is also unlikely that changes in the size and composition of the thiol pool are directly explaining the phytotoxicity of H_2_S. Enhanced glutathione levels may potentially deregulate the activity of various enzymes and cysteine may possibly react with metabolic aldehydes (Rennenberg, 1981). Nevertheless, various plants (especially monocots) can tolerate strongly elevated shoot cysteine and glutathione levels in the presence of H_2_S without any negative impact on biomass production, even after prolonged H_2_S exposure (De Kok, 1989, 1990; Stulen et al., 1990, 2000). In this perspective, it is noteworthy that enhanced glutathione levels may actually also be beneficial for plants. Glutathione has antioxidant properties and elevated glutathione levels may consequently increase the tolerance of plants to environmental stress (Noctor et al., 2012). However, the physiological significance of increased glutathione levels for plant stress protection remains elusive. Exposure of Chinese cabbage (*Brassica pekinensis*) to copper reduced biomass production (Shahbaz et al., 2014). The copper tolerance of Chinese cabbage was not affected by fumigation with subtoxic H_2_S levels, even though this strongly enhanced water-soluble non-protein thiol levels (Shahbaz et al., 2014). Furthermore, in maize, the increased thiol levels upon subtoxic H_2_S fumigation could not counteract the negative impact of salinity on growth (Ausma et al., 2017). Finally, exposure to subtoxic H_2_S levels did not affect the freezing tolerance of the foliage of winter wheat (Stuiver et al., 1992).

Besides affecting water-soluble non-protein thiol levels, H_2_S exposure may affect the levels of other sulfur metabolites. For instance, in onion (*Allium cepa*) H_2_S exposure resulted in an accumulation of secondary sulfur compounds (*viz.* alliins or their precursors) in the shoot (Durenkamp et al., 2005). Similar to the thiol accumulations, onion could show strongly elevated levels of these compounds without any negative growth effects (Durenkamp et al., 2005). Thale cress accumulated thiosulfate in the shoot upon exposure to a high H_2_S level (1 μl L^−1^; Birke et al., 2015). However, by contrast, exposure to a lower H_2_S concentration (0.25 μl L^−1^) did not affect the thiosulfate content of several crop species (Buwalda et al., 1993). This suggests that thiosulfate formation is likely also not connected to the phytotoxicity of H_2_S. In some species, H_2_S exposure resulted in increased shoot sulfate levels (Durenkamp and De Kok, 2002, 2004, 2005; Durenkamp et al., 2005, 2007). Sulfate accumulation presumably occurs in the vacuole and is, therefore, probably likewise not involved in H_2_S toxicity (Durenkamp et al., 2005). Enhanced sulfate levels upon H_2_S exposure may be caused by the degradation of excessively accumulated organic sulfur compounds and/or by the oxidation of absorbed H_2_S (via sulfide oxidases or via non-enzymatic processes; Durenkamp et al., 2005). Alternatively, it may be caused by a poor regulatory control of H_2_S over the uptake and assimilation of sulfate (Durenkamp et al., 2005). However, in a plethora of tested plants, *viz.* common duckweed (*Lemna minor*), spinach, tobacco (*Nicotiana tabacum*), and *Brassica* species, there was a good regulatory control of H_2_S over the metabolism of sulfate: in these plants H_2_S absorbance downregulated sulfate uptake and assimilation (Brunold and Erismann, 1975; Herschbach et al., 1995a,b; De Kok et al., 2002). The background of this phenomenon has most extensively been investigated in *Brassica* seedlings.

## Impact of H_2_S on the Uptake and Assimilation of Sulfate in *Brassica*

The genus *Brassica* contains various agriculturally relevant crops. *Brassica* species are characterized by a high content of secondary sulfur compounds (*viz.* glucosinolates), leading to an extraordinary high sulfur demand for growth (Aghajanzadeh et al., 2014, 2015). Additionally, *Brassica* species are characterized by a high tolerance to atmospheric H_2_S. Biomass production of *Brassica* seedlings was only significantly reduced upon prolonged exposure to ≥0.4 μl L^−1^ H_2_S (Westerman et al., 2000a).

Similar to observations for other species, in *Brassica*, cysteine and glutathione levels increased in the shoot upon H_2_S exposure (at 0.8 μl L^−1^ H_2_S, approximately 12- and 3.5-fold, respectively), whereas their content in the root was hardly affected (De Kok et al., 2000; Buchner et al., 2004; Koralewska et al., 2008). In *Brassica*, H_2_S fumigation did additionally hardly affect the composition and size of the glucosinolate pool (Aghajanzadeh et al., 2014, 2015).

**Figure 2:**
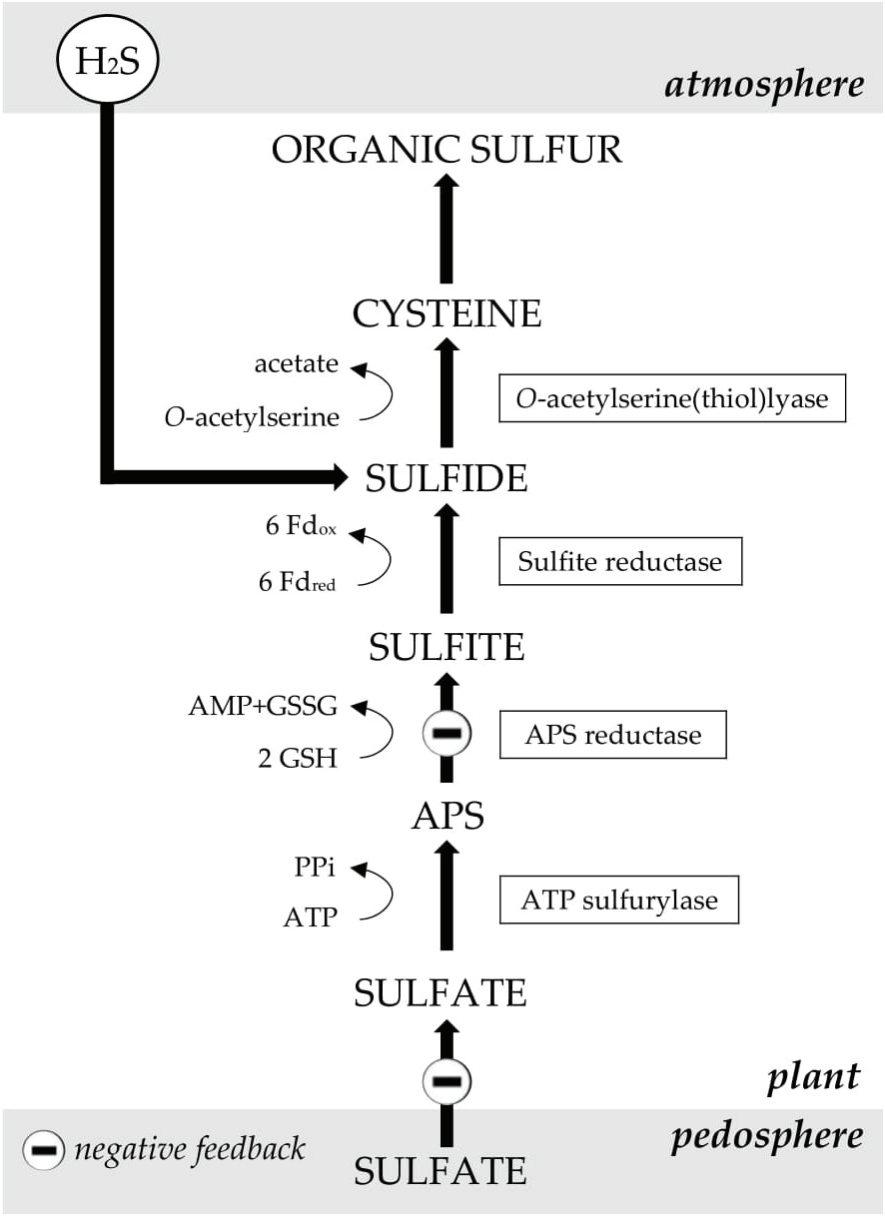
The metabolism of sulfate and the presumed sites in *Brassica* at which H_2_S absorbance downregulates sulfate uptake and assimilation (at an ample sulfate supply). APS: adenosine 5′-phosphosulfate; Fd_red_, Fd_ox_: reduced and oxidized ferrodoxin; GSH, GSSG: reduced and oxidized glutathione. Figure modified after De Kok et al. (2009).

*Brassica* species may switch from sulfate taken up by the root to H_2_S taken up by the shoot as sulfur source (De Kok et al., 1997, 2000; Westerman et al., 2000a,b; Buchner et al., 2004; Koralewska et al., 2008). If these species were grown on a sulfate-rich medium, H_2_S exposure caused a partial downregulation in the activity of transporters involved in root sulfate uptake and sulfate distribution to the shoot (Figure 2; De Kok et al., 1997, 1998, 2000; Westerman et al., 2000a,b; Buchner et al., 2004; Koralewska et al., 2008). This downregulation occurred within a few days after the onset of H_2_S exposure and the extent of the downregulation was dependent on the applied H_2_S concentration (Westerman et al., 2000a). However, in curly kale, this downregulation was never greater than 60% (reached at 0.2 μl L^−1^ H_2_S; Westerman et al., 2000a). Higher repressions appeared unnecessary, since in curly kale the organic sulfur fraction constitutes approximately 60% of total sulfur, whereas sulfate makes up for the other 40% (De Kok et al., 2000; Westerman et al., 2000a). Consequently, the total sulfur and sulfate content of *Brassica* were usually hardly affected by H_2_S fumigation (De Kok et al., 1997, 2000; Westerman et al., 2000a,b; Buchner et al., 2004; Koralewska et al., 2008).

Besides repressing sulfate uptake, H_2_S exposure may lower sulfate assimilation rates (Figure 2). Briefly, during sulfate assimilation, which is located in plastids of both the root and shoot, sulfate is first converted to adenosine 5′-phosphosulfate (APS) by the enzyme ATP sulfurylase (ATPS; Hawkesford and De Kok, 2006). The majority of APS is subsequently reduced to sulfite by the enzyme APS reductase (APR), which controls the rate of sulfate assimilation (Hawkesford and De Kok, 2006). Sulfite is, in-turn, reduced to sulfide by the enzyme sulfite reductase (SIR; Hawkesford and De Kok, 2006). Finally, sulfide is incorporated in the amino acid cysteine via a reaction with *O*-acetylserine (OAS), catalyzed by OAS-TL (Hawkesford and De Kok, 2006). In *Brassica*, exposure to H_2_S decreased both the activity and expression of the enzyme APR in shoots and roots (up to 80% at 0.8 μl L^−1^ H_2_S in curly kale; Westerman et al., 2001; De Kok et al., 2002; Aghajanzadeh et al., 2016). The expression and activity of the other enzymes involved in sulfate assimilation *viz.* ATPS, SIR, and OAS-TL were usually hardly affected by H_2_S exposure (De Kok et al., 2000; Stuiver and De Kok, 2001; Westerman et al., 2001).

Evidently, in *Brassica*, there is a good regulatory control of H_2_S over the utilization of sulfate. However, the shoot-to-root signals via which H_2_S downregulates sulfate utilization remain elusive. Though it was proposed that glutathione, sulfate, and/or compounds from nitrogen and carbon metabolism (e.g., amino acids and carbohydrates) may be signal compounds, in *Brassica*, there were no clear correlations between the levels of these metabolites, the activity of the sulfate transporters and the activity of APR (Westerman et al., 2000a; Koralewska et al., 2008; Shahbaz et al., 2014). Yet, it was evident that root sulfate uptake in *Brassica* is strongly controlled by the sink capacity (*viz.* the sulfur status) of the shoot (Koralewska et al., 2009). Furthermore, it was clear that H_2_S presence downregulates sulfate uptake via transcriptional and posttranscriptional mechanisms (Koralewska et al., 2008). Decreases in sulfate uptake capacity upon H_2_S fumigation did not directly correlate with decreases in the expression of sulfate transporter 1;2 (Sultr1;2), which is in *Brassica* – at an ample sulfate supply – the main transporter responsible for root sulfate uptake (Koralewska et al., 2008).

When *Brassica* plants are deprived of sulfur, they induce multiple responses enabling an enhanced sulfur use efficiency. Sulfur deprivation rapidly induced the expression of transporters involved in root sulfate uptake, the transport of sulfate to the shoot and the vacuolar exchange of sulfate (Koralewska et al., 2007, 2008). At sulfate-sufficient conditions, generally only Sultr1;2 is responsible for root sulfate uptake (Buchner et al., 2004). However, sulfur deprivation resulted not only in an enhanced expression of Sultr1;2 but also in a strongly enhanced expression of sulfate transporter 1;1 (Sultr1;1; Buchner et al., 2004; Koralewska et al., 2007, 2008). The upregulated expression of these two sulfate transporters was accompanied by an increased sulfate uptake capacity of the root (Buchner et al., 2004; Koralewska et al., 2007, 2008). Additionally, sulfur deprivation quickly enhanced the expression and activity of APR in both roots and shoots (Koralewska et al., 2007, 2008; Shahbaz et al., 2014; Aghajanzadeh et al., 2016). Moreover, prolonged sulfur deprivation resulted in an altered shoot-to-root biomass partitioning in favor of that of the root (De Kok et al., 1997; Koralewska et al., 2007, 2008). In the absence of a sulfur supply, these changes were accompanied by decreases in sulfate and thiol content (De Kok et al., 1997; Buchner et al., 2004; Aghajanzadeh et al., 2016).

If sulfur-deprived *Brassica* plants were fumigated with ≥0.06 μl L^−1^ H_2_S, growth rate generally restored to the level of plants grown in the presence of sulfate (De Kok et al., 1997; Westerman et al., 2000a,b; Buchner et al., 2004; Aghajanzadeh et al., 2016). Additionally, the enhanced expression of APR and the sulfate transporters in the shoot was largely alleviated (Buchner et al., 2004; Aghajanzadeh et al., 2016). The levels of sulfate in these plants remained low, but the water-soluble non-protein thiol content of the shoot restored to the level of sulfate-sufficient plants (though the level in the roots remained slightly lower; Buchner et al., 2004; Aghajanzadeh et al., 2016). This confirmed that at atmospheric H_2_S levels ≥0.06 μl L^−1^ foliarly absorbed sulfide could fully replace sulfate taken up by the root as sulfur source for growth (De Kok et al., 1997; Westerman et al., 2000a,b; Buchner et al., 2004; Aghajanzadeh et al., 2016). Nevertheless, atmospheric H_2_S exposure of sulfur-deprived plants had little effect on the expression of APR in the root (Buchner et al., 2004; Koralewska et al., 2008; Shahbaz et al., 2014). Furthermore, the expression of Sultr1;1 and Sultr1;2 as well as the sulfate uptake capacity of sulfur-deprived H_2_S-fumigated plants were similar to those of sulfur-deprived plants (Buchner et al., 2004; Koralewska et al., 2008). In addition, the decrease in shoot-to-root biomass partitioning remained largely unaffected (Koralewska et al., 2008). Apparently, when no sulfate is present in the root environment, there is a poor shoot-to-root signaling for the regulation of sulfate utilization in *Brassica*. This suggests that the uptake and subsequent metabolism of sulfate in *Brassica* are at least partly controlled by the sulfate concentration in the root environment (besides the sulfur status of the plant itself).

## Concluding Remarks

The impact of atmospheric H_2_S on vegetation is paradoxical. On the one hand, H_2_S presence may negatively affect plant growth and survival. On the other hand, plants can use the gas as a sulfur source for growth. H_2_S levels found in polluted regions can significantly contribute to the sulfur demand of plants. There is no relation between the rate of H_2_S metabolism in organic compounds and the H_2_S susceptibility of plants, which suggests that metabolizing H_2_S does not constitute a strategy to detoxify absorbed sulfide. By contrast, there may be a strong relation between the rate of H_2_S metabolism and the rate of sulfate metabolism. The uptake and metabolism of H_2_S may strongly downregulate the uptake and metabolism of sulfate.

Studies with *Brassica* have clarified the background of this downregulation. Simultaneously, these investigations illustrated that H_2_S fumigation may be a useful tool for studying the regulation of sulfur homeostasis in plants. H_2_S exposure induces changes in the expression and activity of enzymes involved in sulfate metabolism. Moreover, it alters the levels of sulfur metabolites. However, at subtoxic H_2_S concentrations, biomass production is not affected. Therefore, at subtoxic H_2_S concentrations, changes in metabolic status are not the consequence of changes in growth (*viz.* the result of growth concentration or dilution), but instead the direct consequence of changes in sulfur utilization. Thus, relating changes in, e.g., metabolite content to changes in enzyme expression and activity can be used to unravel signal transduction pathways that control sulfur homeostasis.

In this way, H_2_S fumigation may not only further clarify the regulation of sulfur metabolism in *Brassica*, but also the regulation of sulfur metabolism in other plants. For instance, it may clarify the regulation of sulfate uptake in monocots, in which Sultr1;1 appears to be mainly responsible for the primary uptake of sulfate (instead of Sultr1;2 as in *Brassica*; Buchner et al., 2010). Moreover, fumigation studies may help to elucidate the regulation of sulfate assimilation in C_4_ plants. Research with the genus *Flaveria*, which contains C_3_ and C_4_ species, indicated that C_4_ species may have a higher demand for reduced sulfur than C_3_ species and that sulfate assimilation in C_4_ species may have shifted to the roots compared to C_3_ species (Gerlich et al., 2018).

The application of subtoxic H_2_S levels may additionally help to clarify the physiological significance of sulfur in plants. Analogous to its function in animal physiology, it has been suggested that endogenous sulfide might also in plants function as a signal molecule that modulates plant immunity, senescence, and various other processes (reviewed by Li et al., 2016; Aroca et al., 2018; Hancock, 2018). However, in several studies that addressed the role of endogenous sulfide, plants were cultivated under suboptimal conditions (e.g., under low light intensities) and/or exposed to relatively high concentrations of sodium hydrosulfide (NaHS). If NaHS is added to a nutrient or tissue incubation solution at neutral pH, it will result in a short-term burst of H_2_S, followed by the release of H_2_S into the atmosphere (Lee et al., 2011). This means that plants are briefly exposed to relatively high H_2_S levels in both the root and shoot environment, which may not only be phytotoxic, but which also may interfere with the regulation of sulfur homeostasis (De Kok et al., 2007; Martin and Maricle, 2015). Evidently, H_2_S fumigation of plants provides another way to study the physiological significance of endogenous sulfide as a signal molecule. Foliar H_2_S application may also be useful to investigate if organic sulfur compounds modulate physiological processes, since upon H_2_S fumigation the content of several of these compounds may be strongly altered.

## Author Contributions

TA and LK have both made substantial, direct, and intellectual contributions to this work. Both approved it for publication.

### Conflict of Interest Statement

The authors declare that the research was conducted in the absence of any commercial or financial relationships that could be construed as a potential conflict of interest.
